# Real-life data on safety and efficacy of autologous stem cell transplantation in elderly patients with multiple myeloma

**DOI:** 10.1007/s00277-018-3528-x

**Published:** 2018-10-27

**Authors:** Carolina Marini, Tânia Maia, Rui Bergantim, Jorge Pires, Eliana Aguiar, José Eduardo Guimarães, Fernanda Trigo

**Affiliations:** 1 Centro Hospitalar de São João, Porto, Portugal; 2 Faculdade de Medicina da Universidade do Porto, Porto, Portugal

**Keywords:** Multiple myeloma, Elderly, Autologous transplantation

## Abstract

Autologous stem cell transplantation (ASCT) is still debatable in treatment of patients over 65 years with multiple myeloma (MM). We performed a retrospective analysis of newly diagnosed MM patients who underwent ASCT between January 2010 and July 2016. A non-transplanted group with similar clinical characteristics, aged 65–70 years old, diagnosed and treated in the same timeline was used for comparison. We analyzed a total of 155 patients, 132 of which underwent ASCT (≤ 65 years, *n* = 103, median 56 years; > 65 years, *n* = 29, median 67 years) and 23 non-transplanted (median 68 years). Conditioning consisted of melphalan 200 mg/m^2^ (MEL200) in younger patients and melphalan 140 mg/m^2^ (MEL140) in half of elderly patients. Stratifying by age, there were no statistically significant differences concerning transplant-related myelotoxicity and non-hematopoietic toxicity; however, elderly patients conditioned with MEL200 had higher needs of transfusional support and more days of intravenous antibiotics. Those patients also had higher needs of transfusional support, higher grade of mucositis (*p* = 0.028), and more days of intravenous antibiotics (*p* = 0.019) than the elderly transplanted with MEL140. Global transplant-related mortality was 3.8%. Survival was not influenced by age. Non-transplanted elderly patients had comparable disease features, and induction response was similar in both groups (before ASCT in the transplanted cohort). Survival of transplanted elderly patients was superior to non-transplanted (OS, 59 months vs 30 months, *p* = 0.037; EFS, 45 months vs 27 months, *p* = 0.014). Selected elderly patients when transplanted have similar disease response and survival as younger patients. A higher dose of melphalan has more toxicity, but it is globally a well-tolerated procedure.

## Introduction

Multiple myeloma (MM) is mainly a disease of the elderly [1, 2]. On the one hand, the lengthening of life expectancy is related to an increase in the incidence of oncological diseases, and on the other hand, the improvement of diagnostic acuity and new therapeutic options on MM has led to a longer survival of elderly patients from median 19 months (in 1973) to 6.1 years (in 2004) [1, 3, 4]. A Mayo Clinic study [2] confirmed a significant increase in cases diagnosed in older age groups when comparing to the 1950s and early 2000s, with the median age at diagnosis of MM increasing from 70 to 74 years, as well as doubling the proportion of newly diagnosed patients aged 80 years or older.

Over the last two decades, there were significant advances in understanding MM disease biology and in development of several new drugs that allowed a paradigm shift from a palliative intent towards the active management of the disease aiming to prolong event-free survival (EFS) and overall survival (OS) [1]. Nowadays, induction followed by autologous stem cell transplantation (ASCT) after high-dose melphalan conditioning continues the standard treatment for MM patients under the age of 65 years [1, 5–7]. Some studies suggest that age at the time of transplant does not have prognostic significance on outcome after ASCT [8, 9], but its safety and efficacy remain uncertain for patients over that age [9–16].

Aging determines a progressive deterioration of physiological reserves and endurance that may increase morbidity and mortality, jeopardizing the effectiveness of ASCT as part of treatment options in MM. Despite these concerns, studies show there is a blatant benefit in survival for transplanted elderly patients [14, 17–22], even in analysis adjusted to performance status, comorbidities, and disease stage [20] and after the introduction of novel agents for induction chemotherapy [14, 19, 21]. Therefore, it is imperative to evaluate transplant toxicity in an advanced age. Despite lacking randomized data in the elderly population, recent data reveal that ASCT is becoming more used over 65 years old [12, 19], allowing to determine its efficacy and toxicity in the real-life context for this population.

## Objective

The main goal of this analysis is the evaluation of ASCT’s toxicity in elderly patients (> 65 years old) when compared to a younger cohort (≤ 65 years old). Secondary endpoints included the efficacy of ASCT and evaluation of OS and progression-free survival (PFS) in the transplanted.

## Methods

### Patients and risk stratification

Retrospective analysis of 132 MM patients consecutively submitted to ASCT between January 2010 and July 2016, at the Department of Hematology of Centro Hospitalar São João (Porto, Portugal). Patients were stratified by age into two groups: group 1, ≤ 65 years old, and group 2, > 65 years old. Data from 65- to 70-year-old patients diagnosed in this same calendar period who were not transplanted were used for comparison (group 3). Exclusion criteria included the following: plasma cell leukemia or amyloidosis at diagnosis, death during induction therapy in patients with indication for ASCT or during the first line of treatment in patients with no indication for ASCT who did not complete all cycles, patients in palliative care. This center also received patients referred for ASCT in whom diagnosis and induction therapy were performed at a different hospital. For risk stratification, we used the International Staging System (ISS) [23], Durie Salmon Staging System (DS) [24], and fluorescence in situ hybridization (FISH) for deletion 17p, deletion 1p, gain of 1q, deletion 13q, deletion 16q, deletion 14q, and translocations *t*(4;14), *t*(11;14), and *t*(14;16). The threshold cytogenetics abnormalities were set at 10%.

### Transplant eligibility

Indication for ASCT followed our center protocol in which eligibility criteria include a performance status (PS) ECOG ≤ 2 and no significant comorbidities, evaluated by echocardiogram, respiratory functional studies, and also daily life performance information reported by patient’s attending physician.

### Induction therapy and stem cell mobilization

Patients received two to seven cycles of induction therapy before stem cell mobilization. Induction therapy was applied according to the local protocol of our center and referring institutions. Peripheral hematopoietic progenitor cells were mobilized with cyclophosphamide (4 g/m^2^) and granulocyte colony-stimulating factor (G-CSF) 10 mcg/kg twice a day or G-CSF alone if patients had less than complete response or complete response after induction therapy, respectively.

### Conditioning for transplant

Conditioning regimen consisted of melphalan at high dose 200 mg/m^2^ (MEL200) or with reduced dose 100 or 140 mg/m^2^ (rMEL) according to the presence of organ dysfunctions (creatinine clearance ≤ 40 mL/min or subjective evaluation of the patient’s ability to receive high-dose melphalan). Prophylactic care included antiviral and antifungal prophylaxis, thromboprophylaxis before thrombocytopenia, and Caphosol® for mucositis prophylaxis.

### Assessment of transplant-related toxicity

For transplant safety assessment, data were collected on hematologic toxicity (transfusion needs, time in days until peripheral neutrophil, and platelet count recovery), mucositis (grade, use and duration in days of intravenous morphine), and infection (number of days of fever, antibiotics used, days under antibiotics), days of hospitalization, and need for intensive care during the transplant procedure. Engraftment was defined by absolute neutrophil count (ANC) ≥ 0.5 × 109/L for three consecutive days, and platelet count ≥ 20 × 109/L unsupported by platelet transfusions in the last 7 days. Treatment-related mortality (TRM) was defined as death during the first 100 days after ASCT.

### Assessment of complications among elderly patients

Description and comparison of complications according to Common Terminology Criteria for Adverse Events (CTCAE) [25] in elderly patients, transplanted or not, in the first 12 months after beginning treatment. Inpatient days due to complications accounted for excess days in treatment-related hospitalizations and complications severe enough to require hospitalization.

### Assessment of treatment efficacy and survival

Treatment response was assessed according to the IMWG guidelines [26]. It was carried out after induction therapy in all groups and 100 days after ASCT in transplanted patients.

Evaluation of overall survival (OS) and event-free survival (EFS) was performed by age and transplant status. EFS was defined as the time from ASCT to relapse/progression or death from the disease. OS was defined as the time from ASCT to death from any cause.

### Statistical analysis

Statistical analysis was performed using SPSS® v.20 [27]. Normal distribution was characterized by skewness and kurtosis for psychometric variables and by Shapiro-Wilk and Kolmogorov-Smirnov tests (*p* > 0.01). Regarding EFS, we applied nonparametric tests and survival curves according to the Kaplan-Meyer method with the log-rank test to identify differences between groups. For OS, hazard ratio (HR) was calculated according to Cox regression analysis for age and melphalan dose effect. A confidence interval was defined at 95%.

### Ethics committee analysis

This study has been evaluated and approved by our ethics committee, the Ethics Committee for Health of Centro Hospitalar de São João/Faculty of Medicine of Oporto University.

## Results

### Baseline clinical and laboratory features

Patient’s demographics, disease characteristics, and risk stratification by age group are summarized in Table 1. There was a total of 155 MM patients newly diagnosed in the defined period, 103 of which in group 1, 29 in group 2, and 23 in group 3. By Charlson comorbidity index, non-transplanted elderly patients had significantly higher scores ≥ 3 (*p* = 0.02) when compared to transplanted elderly patients and also higher scores when compared to younger patients (*p* = 0.013). No statistical difference in disease characteristics or staging (*p* > 0.05 in all parameters) was found between the three groups.

**Table 1:** Demographics and disease classification at diagnosis

	≤ 65 years old	ASCT > 65 years old	No ASCT ≥ 65 years old
Age at diagnosis, years (median)	56 (36; 65)	67 (64; 70)	68 (65; 70)
Age at transplant, years (median)	56 (37; 65)	68 (66; 70)	NA
Gender			
Male (*n*)	51 (49%)	21 (72%)	11 (48%)
Female (*n*)	52 (51%)	8 (28%)	12 (52%)
Performance status			
ECOG			
≤ 2 (*n*)	103 (100%)	29 (100%)	19 (82%)
> 2 (*n*)	0	0	4 (18%)
Charlson score			
1–2 (*n*)	86 (83%)	18 (62%)	7 (30%)
3–4 (*n*)	16 (16%)	10 (35%)	11 (48%)
≥ 5 (*n*)	1 (1%)	1 (3%)	5 (22%)
CRAB			
Calcium > 2.75 mmol/L (*n*)	15 (15%)	5 (18%)	3 (13%)
Creatinine > 2 mg/dL (*n*)	11 (11%)	4 (16%)	10 (43%)
Hemoglobin < 10 g/dL (*n*)	38 (37%)	9 (31%)	12 (52%)
Bone disease (*n*)	74 (71%)	21 (72%)	12 (66%)
B2MICRO > 3.5 mg/L (*n*)	40 (43%)	14 (54%)	14 (88%)
Monoclonal component			
IgG (*n*)	54 (52%)	13 (45%)	11 (48%)
IgA (*n*)	19 (18%)	11 (38%)	6 (26%)
Light chains only (*n*)	18 (17%)	4 (14%)	6 (26%)
Other (*n*)	12 (13%)	1 (3%)	0
Staging ISS			
I (*n*)	39 (38%)	7 (24%)	3 (13%)
II (*n*)	32 (31%)	10 (35%)	4 (17%)
III (*n*)	26 (25%)	10 (35%)	13 (56%)
Unknown (*n*)	6 (6%)	2 (6%)	3 (14%)
Cytogenetic risk			
Standard (*n*)	30 (29%)	11 (38%)	4 (17%)
Intermediate (*n*)	24 (23%)	5 (17%)	10 (43%)
High (*n*)	12 (12%)	2 (7%)	2 (9%)
Unknown (*n*)	37 (36%)	11 (38%)	7 (31%)
BM plasma cells (median)	18 (0; 80)	10 (0.5; 80)	15 (1; 86)

Charlson score is calculated at time of diagnosis; CRAB is acronym for calcium, renal, anemia, bone, for clinical classification of MM [28]; BM plasma cells is median percentage of plasma cells in bone marrow at diagnosis, by immunophenotype. Cytogenetic risk is defined by Mayo Stratification of Myeloma and Risk Adapted Therapy consensus guidelines 2013 (mSMART) [29]

### Treatment, response to induction, and mobilization

Regarding treatment protocol selection (Table 2), there was no statistical difference in choice of novel agents according to age (*p* = 0.17) or ASCT status (*p* = 0.58) (Table 1). Concerning treatment response after induction, 73% of younger transplanted patients had at least very good partial response (VGPR) as well as 69% of the elderly transplanted and 65% of the elderly non-transplanted patients (Table 2). Despite these differences, it was not statistically relevant, either in the transplanted group (*p* = 0.72) or in the non-transplanted group (*p* = 0.77) when compared to its age-adjusted cohort. Considering transplanted patients, age did not correlate to the selection of mobilization regimen (*p* = 0.22), to the number of collected PBPCs (*p* = 0.09), or to the number of apheresis for appropriate collection (*p* = 0.55).

**Table 2:** Therapy characteristics and response to induction therapy

	≤ 65 years old	ASCT > 65 years old	No ASCT ≥ 65 years old
Induction chemotherapy			
Bortezomib-based (*n*)	71 (69%)	22 (76%)	15 (65%)
IMiD-based (*n*)	3 (3%)	3 (10%)	4 (17%)
Bortezomib + IMiDs (*n*)	27 (26%)	4 (14%)	3 (13%)
Neither (*n*)	2 (2%)	0	1 (5%)
Response to therapy			
CR (*n*)	29 (28%)	7 (24%)	8 (35%)
VGPR (*n*)	46 (45%)	13 (45%)	7 (30%)
PR (*n*)	27 (26%)	9 (31%)	5 (22%)
Refractory/stable (*n*)	1 (1%)	0	3 (13%)
> 2 lines of treatment for better response (*n*)	13 (12%)	6 (21%)	3 (13%)
Time to transplant (median months)	8 (3; 21)	9 (4; 20)	NA
Mobilization regimen			
HD-Cy + GCSF (*n*)	78 (76%)	25 (86%)	NA
G-CSF only (*n*)	25 (24%)	4 (14%)	NA
CD34+ collected (median × 10^6^/kg)	6.5 (2.2; 35)	5.0 (1.6; 19)	NA
Number of apheresis ≥ 3	23 (22%)	8 (27%)	NA

*IMiDs*, immunomodulatory drugs; *HD-Cy*, high-dose cyclophosphamide; *G-CSF*, granulocyte colony-stimulating factor

### Engraftment and transplant-related toxicity

Evaluation of hematologic toxicity according to age subgroups (Table 3) revealed that elderly patients had the same median days of aplasia as the younger cohort. Concerning non-hematopoietic toxicity, there were no significant differences in all parameters, namely infection and mucositis. This was also verified when adjusting to the dose of melphalan.

**Table 3:** Transplant-related toxicity by age, the dose of melphalan and in the elderly group

	≤ 65 vs > 65	> 65: rMEL vs MEL200	MEL200: ≤ 65 vs > 65
Myelotoxicity			
Aplasia (median days)	12 vs 12 (*p* 0.55)	12 vs 11 (*p* 0.025)	12 vs 11 (*p* 0.051)
PLT recovery (median days)	12 vs 12 (*p* 0.74)	12 vs 12 (*p* 0.11)	12 vs 12 (*p* 0.43)
PLT support (median units)	1 vs 2 (*p* 0.32)	1 vs 2 (*p* 0.20)	1 vs 2 (*p* 0.06)
RBC support (median units)	0 vs 0.5 (*p* 0.32)	0 vs 1 (*p* 0.17)	0 vs 1 (*p* 0.06)
Non-hematopoietic toxicity—infection			
Fever (median days)	2 vs 2 (*p* 0.86)	1 vs 2 (*p* 0.29)	2 vs 2 (*p* 0.30)
CRP (median mg/L)	147 vs 139 (*p* 0.71)	156 vs 129 (*p* 0.52)	145 vs 129 (*p* 0.45)
Antibiotics (median number)	2 vs 2 (*p* 0.75)	1 vs 2 (*p* 0.17)	2 vs 2 (*p* 0.32)
Antibiotics (median days)	9 vs 7 (*p* 0.20)	7 vs 11 (*p* 0.019)	9 vs 11 (*p* 0.21)
Non-hematopoietic toxicity—mucositis			
Grade (median)	III vs III (*p* 0.55)	II vs III (*p* 0.028)	III vs III (*p* 0.09)
IV morphine (% who need)	55% vs 41% (*p* 0.18)	28% vs 63% (*p* 0.12)	56% vs 63% (*p* 0.75)
IV morphine (median days)	3 vs 0 (*p* 0.13)	0 vs 3 (*p* 0.08)	3 vs 3 (*p* 0.61)

However, when assessed by the dose of melphalan solely in the elderly group, older patients conditioned by MEL200 seem to have fewer days until neutrophil recovery but with a greater need for transfusion support. These patients had more mucositis (grade and need of support) and more days of antibiotics than the ones conditioned by rMEL. Five patients had to be admitted to intensive care unit (3 under 65 years old and 2 over 65 years old), four of them due to septic shock, and one patient for stroke and bronchiolitis obliterans with organizing pneumonitis. The median inpatient days were 21 (range 15 to 91), and there were no differences between groups (*p* = 0.19). Charlson comorbidity index did not affect transplant-related toxicity, either by age or by the dose of melphalan.

### Non-transplanted patients

Evaluation of complications in transplanted and non-transplanted elderly patients (Table 4) revealed that transplanted patients had more incidence of complications (*p* = 0.02) and significantly more inpatient days due to these complications (*p* = 0.04). Infection was the most frequent complication, accounting for 40% in transplanted patients and 48% in non-transplanted patients. Regarding severity, transplanted patients had more grade 3–4 complications (*p* = 0.043).

**Table 4:** Complications in elderly patients, transplanted or not

	ASCT > 65 years old	No ASCT ≥ 65 years old
Median number of complications in 1 year	4 (0; 6)	2 (0; 7)
Median number of inpatient days due to complications	8 (0; 50)	0 (0; 53)
Type of complications by number of patients		
Neuropathy (*n*)	9 (31%)	5 (22%)
Thrombotic (*n*)	2 (7%)	3 (13%)
Hemorrhagic (*n*)	2 (7%)	1 (4%)
Infection (*n*)	28 (96%)	17 (74%)
Mucositis (*n*)	24 (82%)	0
Others (*n*)	5 (17%)	9 (39%)
Grade of complications by number of events		
Number of events (*n*)	102	55
Grade 1–2 (*n*)	54 (53%)	42 (76%)
Grade 3–4 (*n*)	48 (47%)	13 (24%)

In the type of complications, the category “Others” includes cardiac, hepatic, endocrine, and cutaneous toxicities

### Response at day 100 and survival

**Graph 1:**
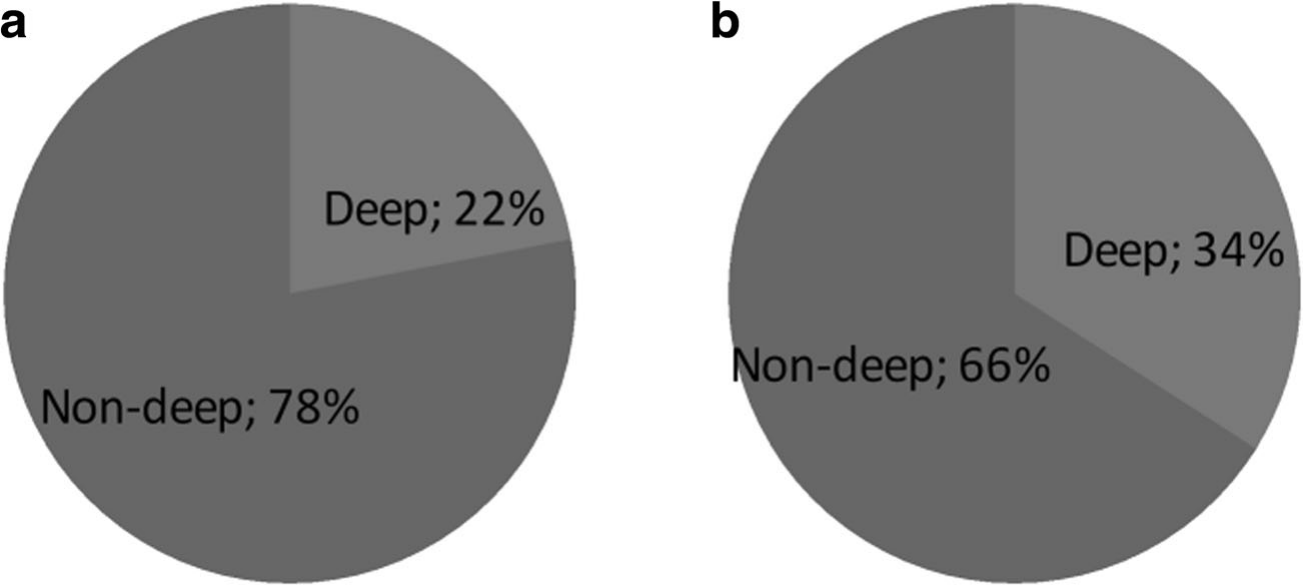
Deepening of response after transplant according to age. **a** Transplanted patients ≤ 65 years old; **b** transplanted elderly patients

After induction therapy, CR was achieved in 27% of transplanted patients. High-dose chemotherapy and ASCT increased the CR rate to 51%. Response at day 100 post-transplant (Table 5) was significantly better than response after induction therapy (*p* < 0.01), with no relation to age. Approximately, a quarter of younger patients and a third of elderly patients had improvement in depth of post-transplant response (Graph 1). Autologous transplantation deepened the level of response as highlighted when comparing to age-adjusted non-transplanted patients (*p* = 0.05).

**Table 5:** Conditioning and outcome after transplant

	≤ 65 years old	ASCT > 65 years old	No ASCT ≥ 65 years old
Conditioning			
MEL200 (*n*)	101 (98%)	11 (38%)	NA
MEL140 (*n*)	2 (2%)	15 (52%)	NA
MEL100 (*n*)	0	3 (10%)	NA
Response at day 100			
CR (%)	50 (49%)	17 (59%)	NA
VGPR (%)	39 (38%)	6 (21%)	NA
PR (%)	10 (9%)	4 (14%)	NA
Refractory/stable (%)	1 (1%)	0	NA
Death at day 100 (%)	3 (3%)	2 (6%)	NA
Mortality (*n*)	21 (19%)	10 (34%)	13 (56%)
MM progression (*n*)	20	7	9
Non-MM related (*n*)	1	3	4
Disease status at last follow-up			
CR (*n*)	40 (49%)	13 (68%)	2 (20%)
VGPR (*n*)	23 (28%)	2 (11%)	1 (10%)
PR (*n*)	5 (6%)	3 (16%)	1 (10%)
Refractory/stable (*n*)	14 (17%)	1 (5%)	6 (60%)

Three patients over 65 years had MEL100 as conditioning regimen: one patient for maintaining renal insufficiency after induction therapy; one patient for reduced number of cells for infusion (1.6 × 10^6^ CD34+ cells/kg); and another patient for subjective evaluation of frailty. Two younger patients had conditioning with MEL140, both for persistent creatinine clearance ≤ 40 mL/min

Five patients died during the transplant procedure (*n* = 3) or the first 100 days after ASCT (*n* = 2) resulting in a TRM of 3.8%, all deaths related to infectious complications.

**Graph 2:**
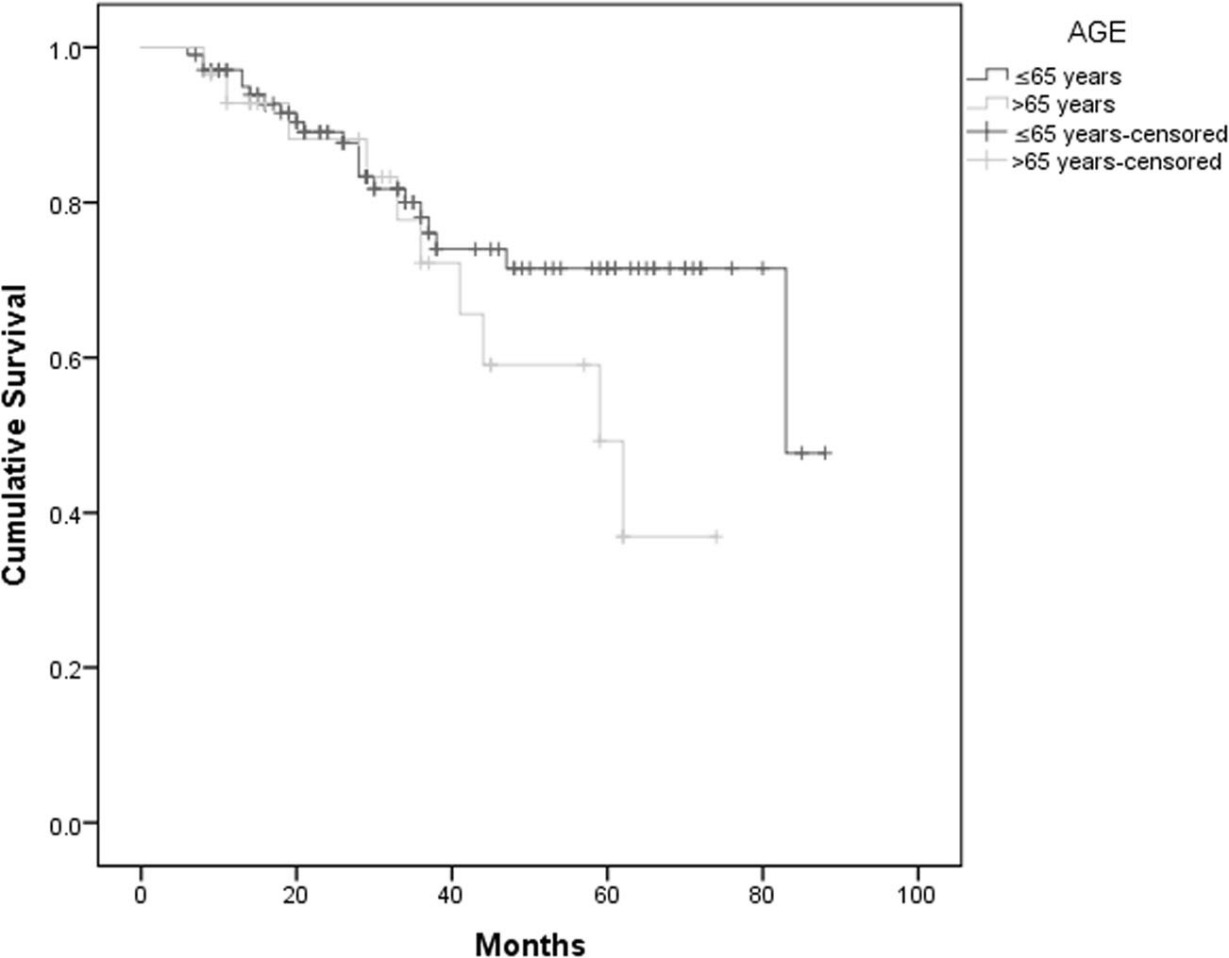
OS according to age, median follow-up 30 months. Median 83 months in patients < 65 years old and 59 months in elderly patients (*p* = 0.15)

**Graph 3:**
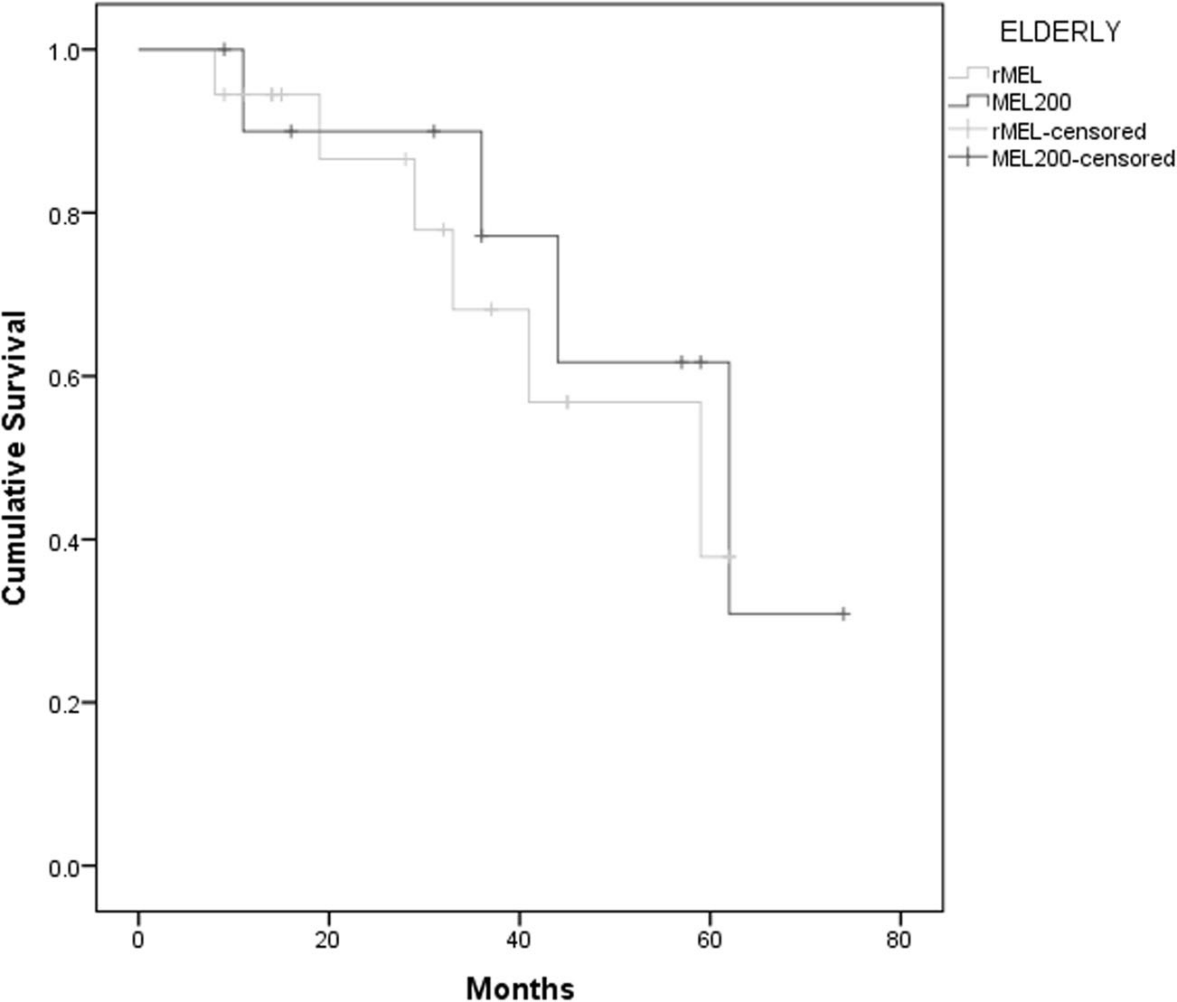
OS according to the dose of melphalan in the elderly group, median follow-up 30 months. Median 59 months in patients conditioned with reduced doses of melphalan vs 62 months in patients conditioned with high doses of melphalan (*p* = 0.73)

**Graph 4:**
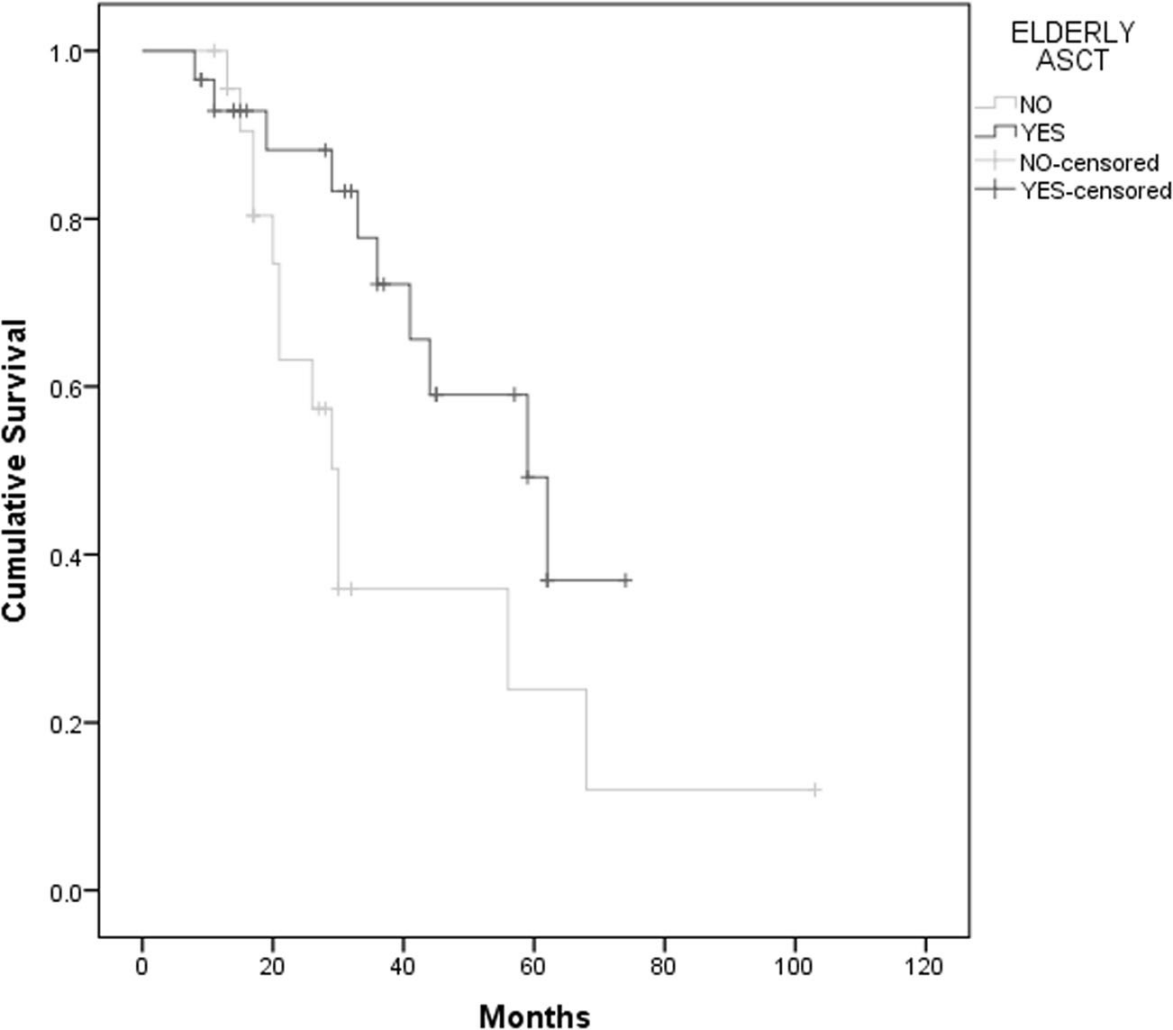
OS of elderly patients according to transplant status, median follow-up 30 months. Transplanted group with a median of 59 months vs 30 months in non-transplanted group (*p* = 0.037)

Data on progression and survival status were collected in September 2016 with a median follow-up of 30 months. Elderly patients had a median EFS of 45 months vs 59 months in younger patient group (*p* = 0.63), with no difference in OS (HR 1.73, CI 0.81–3.70, *p* = 0.15) (Graph 2). Elderly patients conditioned with MEL200 had a median EFS of 62 months vs 45 months in elderly patients treated with a reduced dose of melphalan, however with no statistical significance (*p* = 0.79). There was no effect in OS according to melphalan dose in these patients (HR 0.80, CI 0.22–2.86, *p* = 0.73) (Graph 3). When comparing elderly transplanted patients to non-transplanted patients, there is an essential difference in survival curves. There is an advantage of the transplanted group on EFS with a median time of 45 months vs 27 months on the non-transplanted group (*p* = 0.014). This advantage remains significant on OS (Graph 4).

## Discussion

In our analysis, elderly patients have more toxicity with MEL200 when comparing to reduced doses of melphalan, yet these are manageable complications with the current supportive standard of care. Complications included increased demand for transfusions, support for mucositis, and need for antibiotics for infection control. It is noteworthy that elderly patients treated with MEL200 had less median days of aplasia, which may seem contradictory. Nonetheless, these patients received more supportive measures (higher number of transfused platelet units and erythrocyte concentrates) which may indicate a slower global recuperation. Non-transplanted elderly patients had fewer hospitalizations due to complications, and most of them had low severity, preserving ambulatory management of these patients which is a general aim when handling patients with no curative intent.

There was no excess transplant-related mortality (TRM) in elderly patients and inpatient days were the same regardless of age despite higher toxicity, reinforcing there was an adequate control of complications. Even though autologous transplantation is a standard of care for multiple myeloma, age still plays an important role when pondering this intensification of treatment as questions are raised on the endurance of elderly patients to receive high-dose chemotherapy. It has been demonstrated in several studies that improvement of supportive care provides adequate control of complications [30], reducing discomfort associated with this procedure and even reducing TRM [12, 31]. Cheikh et al. [31] already hypothesized that improvement in supportive care, particularly the use of G-CSF and prophylactic care after intensive chemotherapy, had a positive effect reducing TRM; likewise, Auner et al. [12] observed a marked decrease in mortality throughout the decade 1991–2001, ≤ 2.4% in all age groups, but considerably higher in older than in younger patients; additionally, in another study, Muchtar et al. [30] showed evidence of decreasing infectious complications, transfusion needs, and inpatient days in this same age group from 1998 to 2015, corroborating the favorable impact of supportive care and emphasizing the absence of a biological reason against age to ASCT eligibility.

In recent years, not only the feasibility of ASCT in the elderly has been disputed, but also its efficacy. Most studies are retrospective analysis [9, 11, 30, 32]; some are even before the era of novel agents as proteasome inhibitors (PI) and immunomodulatory drugs (IMIDs) [31, 33, 34], and others use matched pair comparison [35]. Notwithstanding its limitations, these studies found similar toxicity, TRM, and non-inferiority in EFS, OS, or disease response to ASCT in elderly patients, even when considering a higher age threshold for older cohorts as 70 years [30, 32]. In prospective studies [13, 33, 36], patients aged over 65 years do not have an inferior outcome when compared to younger cohorts, considering ASCT is a safe and effective treatment for elderly and fit MM patients, either before [33] or in the present era of novel induction agents [36], which highlights the impact on global outcome that ASCT represents as a component on treatment algorithm in this group of patients [33]. Data is more challenging, considering that there are no randomized trials appropriate to conclude about ASCT in this particular population with the availability of newer effective drugs, namely monoclonal antibodies and new-generation PI or IMIDs [10, 37]. In our study, response after transplant, EFS, and OS did not differ according to age in transplanted patients. Elderly patients had the same benefit comparing to younger patients, either in deepening of response after autologous transplantation or in survival as EFS and OS were not significantly shorter. More so, when compared to the non-transplanted age-adjusted cohort, patients who were transplanted had a significant improvement in EFS and OS, despite having similar disease features. Even though our results seem favorable to transplanted elderly patients, this study shares some of the limitations mentioned above, specifically regarding its retrospective nature, non-randomization of patients, and the subjective categorization for transplant eligibility of elderly patients.

Another matter of discussion, when considering autologous transplantation in elderly patients, is the ideal dose of Melphalan before ASCT. In our analysis, we can see that elderly patients have more toxicity indeed in all parameters with higher doses of melphalan as in previously published data [9, 33]. There is no consensus in the MM community, as some authors defend the use of an intermediate dose of 100 or 140 mg/m^2^ demonstrating equal efficacy in CR rates, EFS, or OS but with lower toxicity [9, 14, 33, 35, 38] or even lower TRM [39]; other authors are in favor of MEL200 as they did not find any excessive toxicity or worse disease outcomes [11, 32, 40]; and others conclude that reduced doses of melphalan can benefit selected cases, but higher doses are preferred [13, 30]. A recent prospective French study [36] demonstrated that there was no difference in myelotoxicity, infections, TRM, or disease response between MEL200 and MEL140, but it was noticed that patients treated with MEL200 had better EFS rate. In our analysis, even though it was not statistically significant, we also noted a trend to a better EFS rate in MEL200 elderly patients when compared to elderly patients treated with reduced doses of melphalan (62 months vs 45 months). However, once again, this was a retrospective analysis of a small and highly selected group of elderly patients with no standardized approach, which limits its statistical power.

## Conclusion

It is suggested in the several aforementioned studies that age by itself is not a reliable prognostic factor for transplantation eligibility as autologous stem cell transplantation in elderly patients is a well-tolerated procedure with current and proper supportive and prophylactic care, with similar TRM, responses rate, EFS, and OS compared to younger patients. In our study, even after MEL200, elderly patients have clinical benefit with acceptable morbidity presenting similar EFS to the one obtained in younger patients, safeguarding that these are highly selected elderly patients.

Taking into account that optimal management of MM is vital for patient outcome, age should not be considered a major obstacle to transplantation. Eligibility should be based on biological fitness and comorbidities, ideally through geriatric assessment tools and comorbidity scores to avoid subjectivity, which was also an important limitation when analyzing our elderly patients.

Our study reflects real data on managing ASCT in elderly patients, offering them possibility of better results and better survival despite all the limitations we mentioned. From our results and revised published data to date, we may argue that ASCT is an essential step of MM treatment and should be offered as a treatment option regardless of age. However, there is a need to include elderly patients in transplant randomized trials to determine a survival benefit in an era of constant newer and effective drugs available.
